# Electrophilic fatty acid nitroalkenes regulate Nrf2 and NF-κB signaling:A medicinal chemistry investigation of structure-function relationships

**DOI:** 10.1038/s41598-018-20460-8

**Published:** 2018-02-02

**Authors:** Nicholas K. H. Khoo, Lihua Li, Sonia R. Salvatore, Francisco J. Schopfer, Bruce A. Freeman

**Affiliations:** Department of Pharmacology and Chemical Biology, University of Pittsburgh, Pittsburgh, PA 15261 USA

## Abstract

Fatty acid nitroalkene derivatives (NO_2_-FA) activate Nrf2-regulated antioxidant gene expression and inhibit NF-κB-dependent cytokine expression. To better define NO_2_-FA structure-function relationships, a series of 22 new chemical entities (NCEs) containing an electrophilic nitroalkene functional group were synthesized and screened for both Nrf2- and NF-κB activities using luciferase-based assays. The structural variables were acyl chain length (11 to 24 carbons) and position of the electrophilic nitroalkene group. In luciferase-based reporter assays, Nrf2 was maximally activated by omega-12 nitroalkene fatty acids while TNFα stimulated NF-κB-inhibition was maximal for omega-5 nitroalkenes. The top pathway-modulating NO_2_-FAs were a) evaluated for an ability to activate Nrf2-dependent signaling and inhibit NF-κB-dependent inflammatory responses of RAW264.7 cells and b) compared to electrophilic compounds in clinical development. These findings revealed that 8/9-nitro-eicos-8-enoic acid (NCE−**10**) was collectively the most effective NCE and that both the α and ω acyl chain lengths influence nitroalkene activation of Nrf2 and inhibition of NF-κB signaling. This insight will guide development of more effective non-natural homologs of endogenously-detectable fatty acid nitroalkenes as anti-inflammatory and anti-fibrotic drug candidates.

## Introduction

Nitro-fatty acids (NO_2_-FAs) are endogenously formed during metabolism and inflammation through the non-enzymatic reaction of unsaturated fatty acids with secondary products of nitric oxide (^•^NO) and nitrite (NO_2_^−^) oxidation, in particular nitrogen dioxide (^•^NO_2_)^1,2^. In rodents, conjugated linoleic acid (CLA) and NO_2_^−^ supplementation increases the basal levels of nitro-conjugated linoleic acid (NO_2_-CLA) in plasma, urine and tissues as a consequence of gastric formation. Clinical studies in humans have shown that the plasma levels of endogenous NO_2_-FAs can be increased four-fold for several hours after one time oral supplementation with CLA and nitrite (NO_2_^−^) or nitrate (NO_3_^−^)^1^. Additionally, coronary artery ligation (ischemia) and a 30 min reperfusion increased the murine cardiac levels of NO_2_-CLA and nitro oleic acid (OA-NO_2_) from undetectable to 17.3 and 9.5 nM, respectively^3^. These studies support the notion that cell and tissue levels of NO_2_-FAs are modulated by diet and oxidative-inflammatory conditions.

The addition of the nitro group (−NO_2_) to the double bond of unsaturated fatty acid (FA) confers a unique electrophilic reactivity characterized by participating in reversible Michael additions and displaying reaction constants with thiols in the order of 10^3^ M^−1^ s^−1 ^^4^. Due to a hydrophobic character, NO_2_-FAs are stabilized by hydrophobic pockets in plasma albumin, while in cells and tissues reactions with glutathione and protein target cysteines (Cys) predominate, with a minor contribution of histidine adduct formation^5^. Current data supports that these reactions instigate adaptive anti-inflammatory and antioxidant cell signaling responses *in vivo* and *in vitro*^6^. Notably, NO_2_-FAs covalently adduct functionally significant cysteine residues (reacting with Cys273 and 288) of Kelch-like ECH-associated protein (Keap)-1 in the cytoplasm, inducing stabilization of nuclear factor (erythroid-derived 2)-like 2 (Nrf2), nuclear translocation and transactivation of gene signaling. This potently activates the transcription of phase II genes through the antioxidant-response element^7–9^. Nrf2-regulated genes are critical in mediating cell defense against oxidative stress, by expressing antioxidant enzymes that limit inflammation and reactive species (RS)-induced damage.

Fatty acid nitroalkenes also alkylate functionally-significant cysteines of the p65 (Cys 38) and p50 (Cys 62) subunits of nuclear factor kappa B (NF-κB) leading to the inhibition of pro-inflammatory transcriptional activity. This inhibits NF-κB translocation and DNA binding, suppression of vascular cell adhesion molecule 1 expression and the inhibition of monocyte rolling, adhesion and tissue infiltration^10–12^. Moreover, NO_2_-FAs inhibit lipopolysaccharide (LPS)-induced secretion of the pro-inflammatory cytokines interleukin-6, tumor necrosis factor-α (TNFα) and monocyte chemotactic protein 1 (MCP1) in macrophages^10^. Electrophilic NO_2_-FAs inhibit inflammation in both cell culture and pre-clinical models, in part through the ability to nitroakylate p50 and p65 subunits of NF-κB.

A series of 22 new chemical entities (NCEs) were designed to determine whether non-natural electrophilic fatty acid nitroalkenes would also activate Nrf2- and inhibit NF-κB-dependent transcriptional regulatory function. Every NCE contained only one nitroalkene group (an NO_2_ bonded to alkenyl carbons). These NCEs consisted of both nitrated fatty acids and fatty alcohols having varying carbon chain lengths. The activity of all 22 NCEs were tested using cell-based luciferase assay systems for defining the relative activation of the antioxidant response element (ARE), which reflects Nrf2 activity, and inhibition of TNFα-induced NF-κB activation. The activities of the NCEs were compared to the clinically-relevant electrophiles nitro-oleic acid (OA-NO_2_), dimethyl fumarate, and the synthetic oleanane triterpenoids (CDDO-imidazolide and CDDO-methyl ester) (Table 1).

**Table 1:** Electrophilic compounds currently in clinical development or use. The chemical structure, abbreviation used through and total number of carbons are shown in this Table.

Clinically relevant electrophiles	Abbr	^#^of C	−NO_2_ position from ω end	
10-Nitro-octadec-9-enoic acid Nitro-oleic acid	OA-NO_2_	18	9	
2,3-Dimethyl-2-butenedioate; Dimethyl Fumarate	DMF	6	—	
4-Methoxy-4-oxo-2-butenoic acid; Monomethyl Fumarate	MMF	5	—	
1-(2-Cyano-3,12,28-trioxooleana-1,9(11)-dien-28-yl)−1H-imidazole; Bardoxolone imidazolide	CDDO-Im	34	—	
Methyl 2-cyano-3,12-dioxooleana-1,9(11)-dien-28-oate; Bardoxolone methyl	CDDO-me	32	—	

The nitroalkene 10-OA-NO_2_ is now entering three Phase II clinical trials for the treatment of focal segmental glomerulosclerosis, asthma and pulmonary arterial hypertension after successful completion of five Phase 1 clinical studies in humans (NCT: 02127190, 02248051, 02460146, 02547402 and 02313064). Dimethyl fumarate (DMF), the dimethyl ester of fumaric acid, was approved by the Food and Drug Administration in 2013 as an oral drug for treating multiple sclerosis under the trade name Tecfidera^13^. The synthetic oleanane triterpenoid CDDO-methyl ester (known as Bardoxolone methyl) has been investigated clinically for the treatment of chronic kidney disease, pulmonary arterial hypertension and as an anticancer agent in solid tumors and lymphomas^14–17^. These agents are all pleiotropic mediators, due to their broad electrophilic reactivity. Preclinical studies of each of these clinical-stage electrophiles affirm anti-oxidant and anti-inflammatory properties that act through Nrf2 induction and inhibition of NF-κB activity. The current study places the signaling actions of dimethyl fumarate and bardoxolone in the context of various nitroalkene structures.

To improve our understanding of structure-function relationships of fatty acyl nitroalkene derivatives, the electrophilic group was introduced at different positions on the acyl carbon chain on fatty acids and fatty alcohols. The objectives of this study were to determine: (1) nitroalkene structure and function (ARE and NF-κB luciferase activity) relationships by specifically testing whether NCEs activate ARE luciferase activity or inhibit TNFα-induced NF-κB luciferase activity, (2) the effects of carboxylic versus alcohol fatty acyl chain, (3) ARE and NF-κB activity responses to omega terminal nitroalkenes and (4) the relative activities of the NCEs versus OA-NO_2_, DMF and synthetic oleanane triterpenoids. Lastly, the lead NCE candidates, based on the luciferase assay screens, were evaluated for whether luciferase-based transcriptional responses reflected an impact on downstream signaling and alterations in mRNA and protein expression in a cell culture model of inflammatory responses using RAW264.7 macrophages.

## Results

### NCE characterization and purity

All NCEs were synthesized and structurally characterized by ^1^H NMR and LC-MS (structures illustrated in Tables 1 and 2) with a purity greater than 95% for all compounds.

**Table 2:** New chemical entities. The chemical structure and name, identification number, total number of carbons and location of the nitroalkene substituent (an NO_2_ bonded to alkenyl carbons) are shown in this Table.

ID	New Chemical Entity	^#^of C	−NO_2_ position from ω end	
1	9/10-nitro-tetradec-9-enoic acid	14	5/6	
2	10/11-nitro-pentadec-10-enoic acid	15	5/6	
3	9/10-nitro-hexadec-9-enoic acid	16	7/8	
4	10/11-nitro-heptadec-10-enoic acid	17	7/8	
5	6/7-nitro-octadec-6-enoic acid	18	12/13	
6	9/10-nitro-octadec-9-enoic acid	18	9/10	
7	11/12-nitro-octadec-11-enoic acid	18	7/8	
8	7/8-nitro-nonadec-7-enoic acid	19	12/13	
9	10/11-nitro-nonadec-10-enoic acid	19	9/10	
10	8/9-nitro-eicos-8-enoic acid	20	12/13	
11	11/12-nitro-eicos-11-enoic acid	20	9/10	
12	5/6-nitro-eicos-5-enoic acid	20	15/16	
13	12/13-nitro-heneicos-12-enoic acid	21	9/10	
14	13/14-nitro-docos-13-enoic acid	22	9/10	
15	14/15-nitro-tricos-14-enoic acid	23	9/10	
16	15/16-nitro-tetracos-15-enoic acid	24	9/10	
17	9/10-nitro-octadec-9-en-1-ol	18	9/10	
18	10-nitro-undec-10-enoic acid	11	1	
19	11-nitro-dodec-11-enoic acid	12	1	
20	12-nitro-tridec-12-enoic acid	13	1	
21	14-nitro-pentadec-14-enoic acid	15	1	
22	10-nitro-undec-10-en-1-ol	12	1	

### Cytotoxicity of NCEs in each model system

The potential cytotoxicity of all NCEs was first determined in HepG2 ARE reporter cells and HEK293 NK-κB reporter cells. There was no cytotoxicity observed, using the MTT assay, in the HepG2 cells (stably integrated with the ARE) treated with all 22 NCEs for 18 hr up to a maximal concentration of 10 μM compared to media alone (Table 3). The results in Table 3 were normalized to HepG2 cells that had a media change 18 hr prior, with data expressed as a percent of the media control. The vehicle control (DMSO), OA-NO_2_ and DMF did not induce any changes in cell viability whereas there was a significant amount of cell death induced by 1 μM CDDO-Im for 18 hr. A lower concentration of 0.1 μM CDDO-Im for 18 hr did not show any toxicity. These experimental conditions were confirmed using Calcein AM, which measures esterase activity, with identical results (not shown). There was also no cytotoxicity upon co-treatment with the different electrophiles and 500 pg/mL TNFα for 2 hr (Table 4).

**Table 3:** Cytotoxicity screen of stably transfected HepG2 (ARE reporter). Cells were seeded at 2 × 10^4^ cells/well in a 96-well plate overnight and then subsequently treated with 10 μM for all NCE (1–22), OA-NO_2_, DMF or CDDO-Im (0.1 and 1 μM) for 18 hours. Cell viability was determined by the MTT assay. The data was normalized to the media alone treatment (cells with new media) and expressed as a percent. All data represents the means ± SEM of at least three independent experiments (within each individual experiment, there was a minimum of four replicates per treatment). c, p < 0.01 vs DMSO.

Treatment	% control
DMSO	110.9 ± 3.8
1	117.0 ± 4.5
2	116.9 ± 5.2
3	117.3 ± 3.3
4	112.6 ± 3.7
5	113.4 ± 3.7
6	113.1 ± 5.1
7	110.3 ± 4.5
8	115.6 ± 5.5
9	114.9 ± 4.0
10	119.6 ± 3.4
11	117.1 ± 3.9
12	118.8 ± 6.7
13	123.6 ± 6.6
14	126.2 ± 4.4
15	118.4 ± 5.9
16	123.3 ± 4.2
17	127.2 ± 2.1
18	134.4 ± 3.6
19	141.6 ± 4.8
20	137.3 ± 2.5
21	145.3 ± 5.9
22	130.8 ± 2.6
OA-NO_2_	113.0 ± 3.8
DMF	127.7 ± 7.7
0.1 μM CDDO-Im	117.5 ± 3.3
1 μM CDDO-Im	6.2 ± 13.9 c

**Table 4:** Cytotoxicity screen of stably transfected HEK293 (NF-kB reporter). Cells were seeded at 4 × 10^4^ cells/well in a 96-well plate overnight and then subsequently co-treated with 500 pg/mL TNFα and 10 μM for all NCE (1–22), OA-NO_2_, DMF or CDDO-Im (0.1 and 1 μM) for 2 hours. Cell viability was determined by the MTT assay. The data was normalized to the media alone treatment (no TNFα) and expressed as a percent. All data represents the mean ± SEM of at least three independent experiments (within each individual experiment, there was a minimum of four replicates per treatment).

	Treatment	% control
500 pg/mL TNFα +	**media**	87.2 ± 7.1
	**DMSO**	89.2 ± 4.1
	**1**	88.3 ± 0.7
	**2**	83.2 ± 1.9
	**3**	85.9 ± 3.5
	**4**	89.0 ± 2.4
	**5**	92.8 ± 6.4
	**6**	100.4 ± 4.5
	**7**	100.7 ± 5.2
	**8**	93.5 ± 4.5
	**9**	96.6 ± 1.0
	**10**	96.5 ± 2.2
	**11**	104.4 ± 4.4
	**12**	92.9 ± 0.5
	**13**	94.9 ± 6.5
	**14**	94.2 ± 5.0
	**15**	87.2 ± 4.3
	**16**	93.3 ± 5.0
	**17**	103.8 ± 2.1
	**18**	103.9 ± 5.2
	**19**	102.5 ± 5.0
	**20**	100.6 ± 3.1
	**21**	95.2 ± 3.0
	**22**	105.8 ± 2.9
	**OA-NO**_**2**_	91.3 ± 3.0
	**DMF**	92.7 ± 5.4
	**0**.**1** μ**M CDDO-Im**	99.7 ± 7.0
	**1** μ**M CDDO-Im**	101.1 ± 2.4

### NCE ARE activity

**Figure 1:**
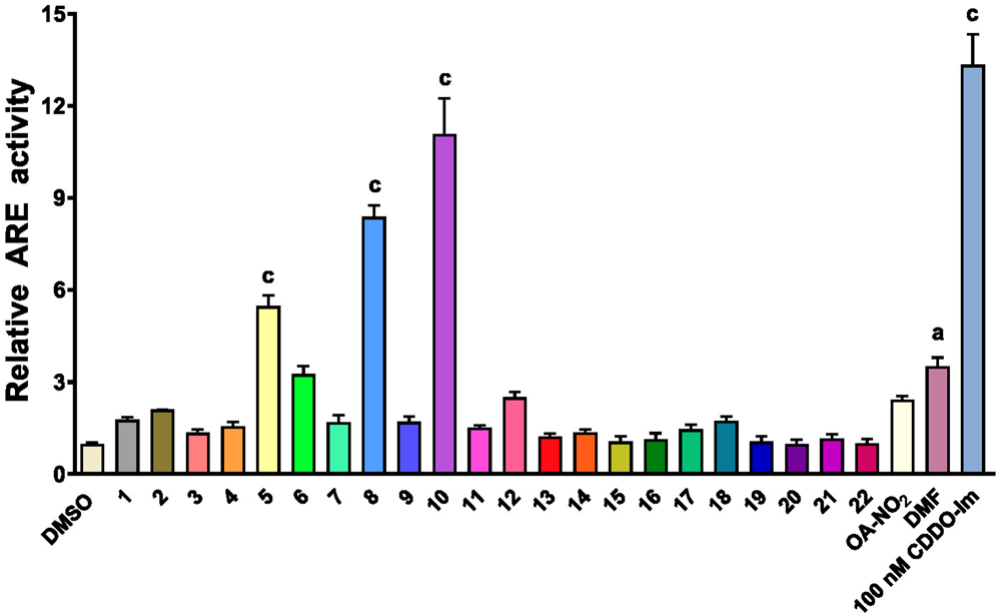
Relative Nrf2/ARE luciferase activity. Stable ARE‐luc HepG2 cells were treated for 18 hours at 10 μM for all NCE (1–22), OA-NO_2_, DMF and 100 nM CDDO-Im. Within an individual experiment, there was a minimum of a n = 3/treatment which was normalized to the media alone treatment (cell media was replaced only). These technical replicates were averaged for each treatment. The values in the bar graph represent mean ± SEM for at least four independent experiments (n = 4–12). Compared to the vehicle (DMSO) treatment group, statistical significance is indicated by: (a) p < 0.05 and (c) p < 0.001.

The luciferase system was first tested using established Nrf2 activators. DMF activated ARE activity (3.52 ± 0.29) slightly greater than OA-NO_2_ at the 10 μM concentration. CDDO-Im potently activated ARE activity (13.3 ± 1.00) at much lower concentrations (100 nM where there was no observed cytotoxicity at 18 hr), compared to 1 μM CDDO-Im in Table 3. Next, the NCEs (10 μM) were tested and there were five NCEs that had greater relative ARE luciferase activities compared to 10 μM OA-NO_2_ (2.40 ± 0.14-fold increase compared to media-treated cells, Fig. 1). These five derivatives were NCE-10, -8, -5, -6 and -12, ranked from highest to lowest, with NCE-10, -8 and -5 all being omega-12 nitro-alkenes. Finally, there was no difference between media alone and vehicle (DMSO).

### NCE inhibition of TNFα-induced NF-κB luciferase activity

**Figure 2:**
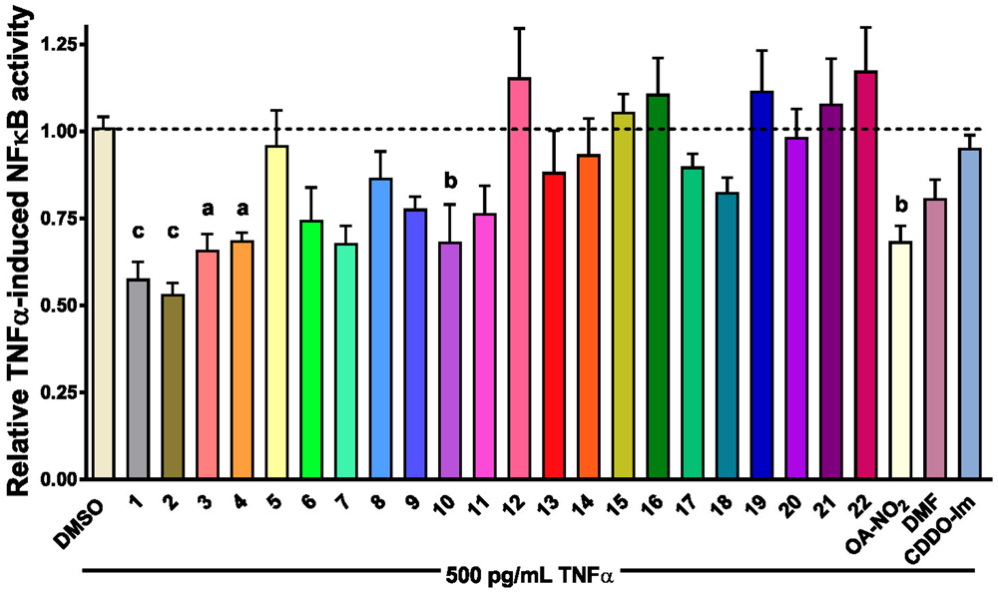
Relative NF-κB luciferase activity. Stable HEK293cells were co-treated for 2 hours with 500 pg/mL TNFα (to induced NF-κB transactivation) and electrophilic compounds (all at a concentration of 10 μM, except 100 nM CDDO-Im). Within an individual experiment, there was a minimum of a n = 3/treatment which was normalized to the 500 pg/mL TNFα treatment with media. These technical replicates were averaged for each treatment. The values in the bar graph represent mean ± SEM for at least four independent experiments (n = 4–10). Statistical significance was defined (see below) using the Tukey posthoc test following one-way ANOVA for all 27 treatments- 22 NCEs, OA-NO2, DMF, CDDO-Im, media and DMSO). Compared to the vehicle (DMSO) treatment group, statistical significance is indicated by: (a) p < 0.05; (b) p < 0.01 and (c) p < 0.001.

Figure 2 shows that six NCEs significantly inhibited TNFα-induced NF-κB luciferase activity at 10 μM compared to TNFα treated cells. All six electrophilic compounds (NCE: 1–4, 10 and OA-NO_2_) inhibited TNFα-induced NF-κB luciferase activity greater than 20%. Out of the top six, NCEs (1 and 2) and OA-NO_2_ were the most potent in inhibiting TNFα-induced NF-κB luciferase activity (p ≤ 0.001 compared to the media-treated cells stimulated with TNFα). The remaining NCEs did not induce statistically significant responses compared to TNFα + media, showing little or no ability to inhibit TNFα-induced NF-κB luciferase activity. NCEs having terminal nitroalkene groups (omega end, NCE: 18–21) and the fatty alcohol nitroalkenes (NCE-17 and -22) failed to inhibit NF-κB luciferase activity. DMF and CDDO-Im did not significantly inhibit TNFα-induced NF-κB luciferase activity.

**Figure 3:**
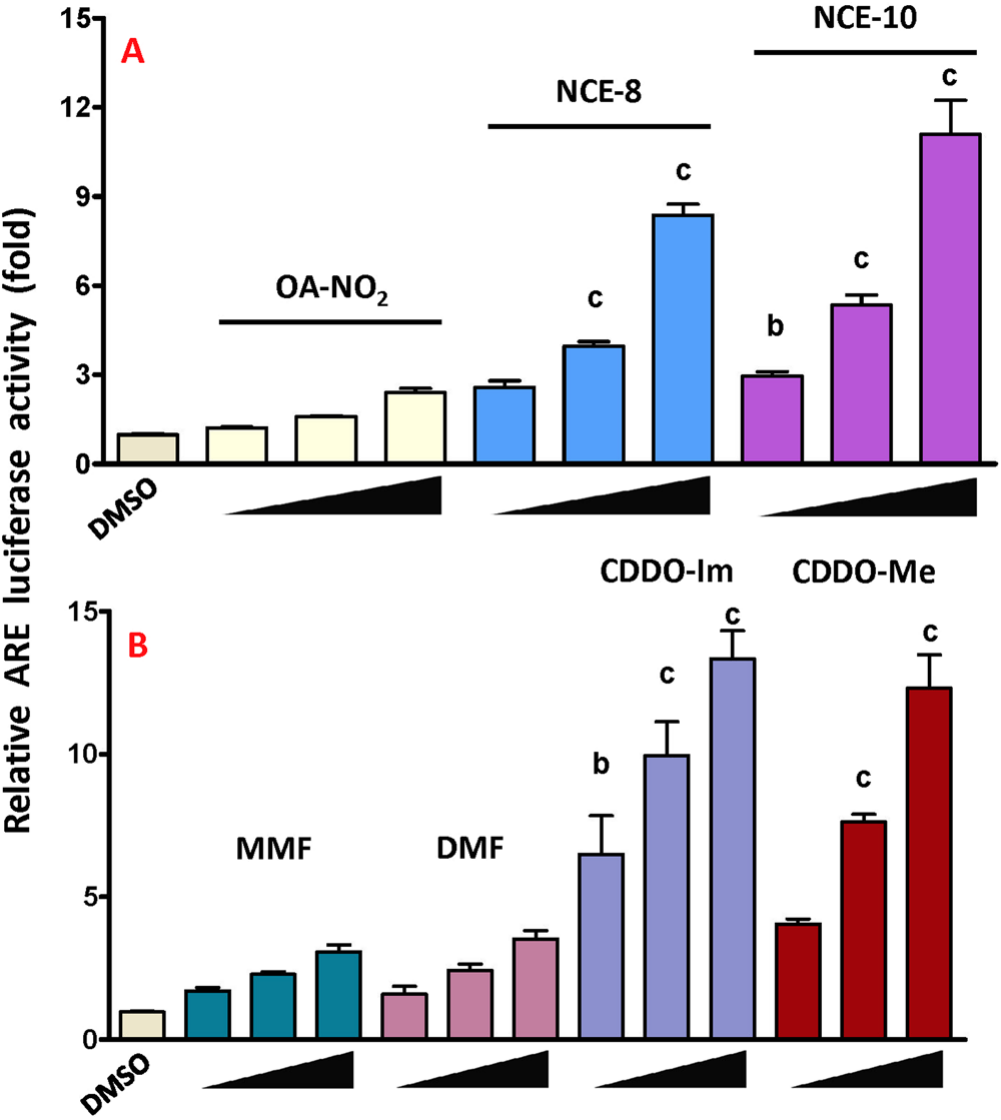
Dose-dependent increase in ARE luciferase activity. Stable ARE‐luc HepG2 cells were treated for 18 hours with increasing concentrations (2.5, 5 and 10 μM) of OA-NO_2_, NCE-8 and NCE-10 in (**A**). Electrophilic compounds currently in clinical development or use that activate ARE were also tested with increasing concentrations at 2.5, 5 and 10 μM for MMF and DMF (to match NCE concentrations) as well as 10, 50 and 100 nM of CDDO-Im and CDDO-Me in (**B**). Within an individual experiment, there was a minimum of an n = 4/treatment which was normalized to the media alone treatment (cell media was replaced only). These technical replicates were averaged for each treatment. The values in the bar graph represent mean ± SEM for at least three independent experiments (n = 3–12). Statistical significance was defined (see below) using the Tukey posthoc test following one-way ANOVA for all 27 treatments- 22 NCEs, OA-NO2, DMF, CDDO-Im, media and DMSO). Compared to the vehicle (DMSO) treatment group, statistical significance is indicated by: (a) p < 0.05; (b) p < 0.01 and (c) p < 0.001.

**Figure 4:**
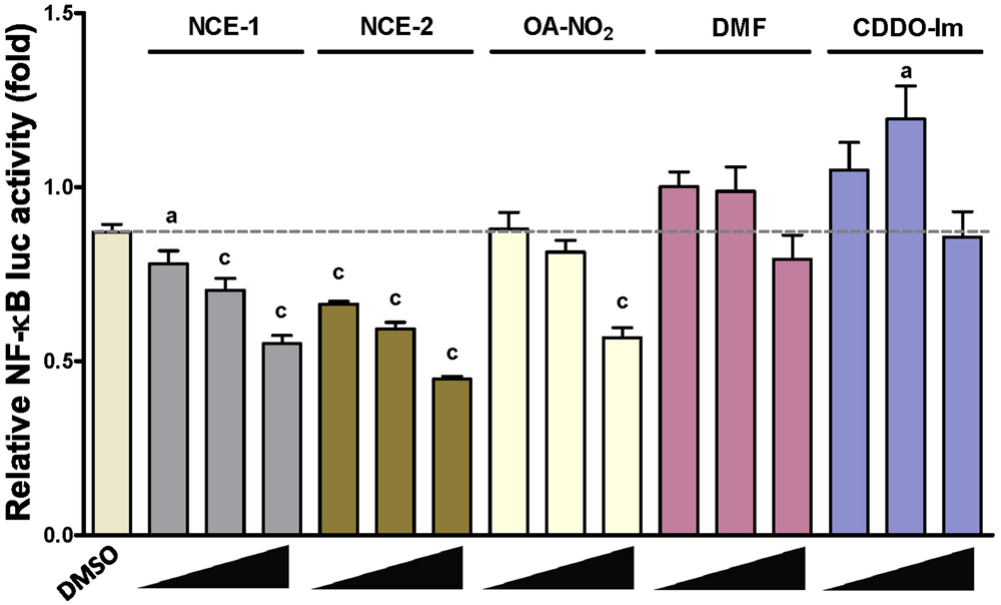
Dose-dependent inhibition of TNFα-induced NF-kB luciferase activity. Stable HEK293cells were co-treated for 2 hours with 100 pg/mL TNFα (to induced NF-κB activity) and with increasing concentrations at 2.5, 5 and 10 μM for all electrophilic compounds with the exception of CDDO-Im (10, 50 and 100 nM). Within an individual experiment, there was a minimum of an n = 4/treatment which was normalized to the 100 pg/mL TNFα treatment in media. The values in the bar graph represent mean ± SEM for at least three independent experiments (n = 3–6). Compared to the vehicle (DMSO) treatment group, statistical significance is indicated by: (a) p < 0.05 and (c) p < 0.001.

The top ARE-activating (8 and 10) and NF-κB-inhibiting (1 and 2) NCEs were further evaluated with a dose response curve. Stable ARE-luc HepG2 cells were treated for 18 hr with increasing concentrations (2.5, 5 and 10 μM) of NCE-8 and -10 compared with OA-NO_2_ (Fig. 3A). A dose response was also performed for DMF and mono methyl fumarate (MMF) as well as 10, 50 and 100 nM of CDDO-Im and CDDO-Me in Fig. 3B. Stable NF-κB-luc HEK293 cells were stimulated using a lower concentration of 100 pg/mL TNFα to avoid an overwhelming inflammatory response. Cells were co-incubated with increasing concentrations of NCEs, OA-NO_2_, DMF and CDDO-Im for 2 hr (Fig. 4). There was a significant dose-dependent inhibition of NK-κB luciferase activity with NCE-1 and -2 and a lack of an effect by DMF and CDDO-Im (Fig. 4). Also, MMF and CDDO-me failed to inhibit TNFα-stimulated NF-κB luciferase activity (not shown).

### Gene expression responses to NCEs

**Figure 5:**
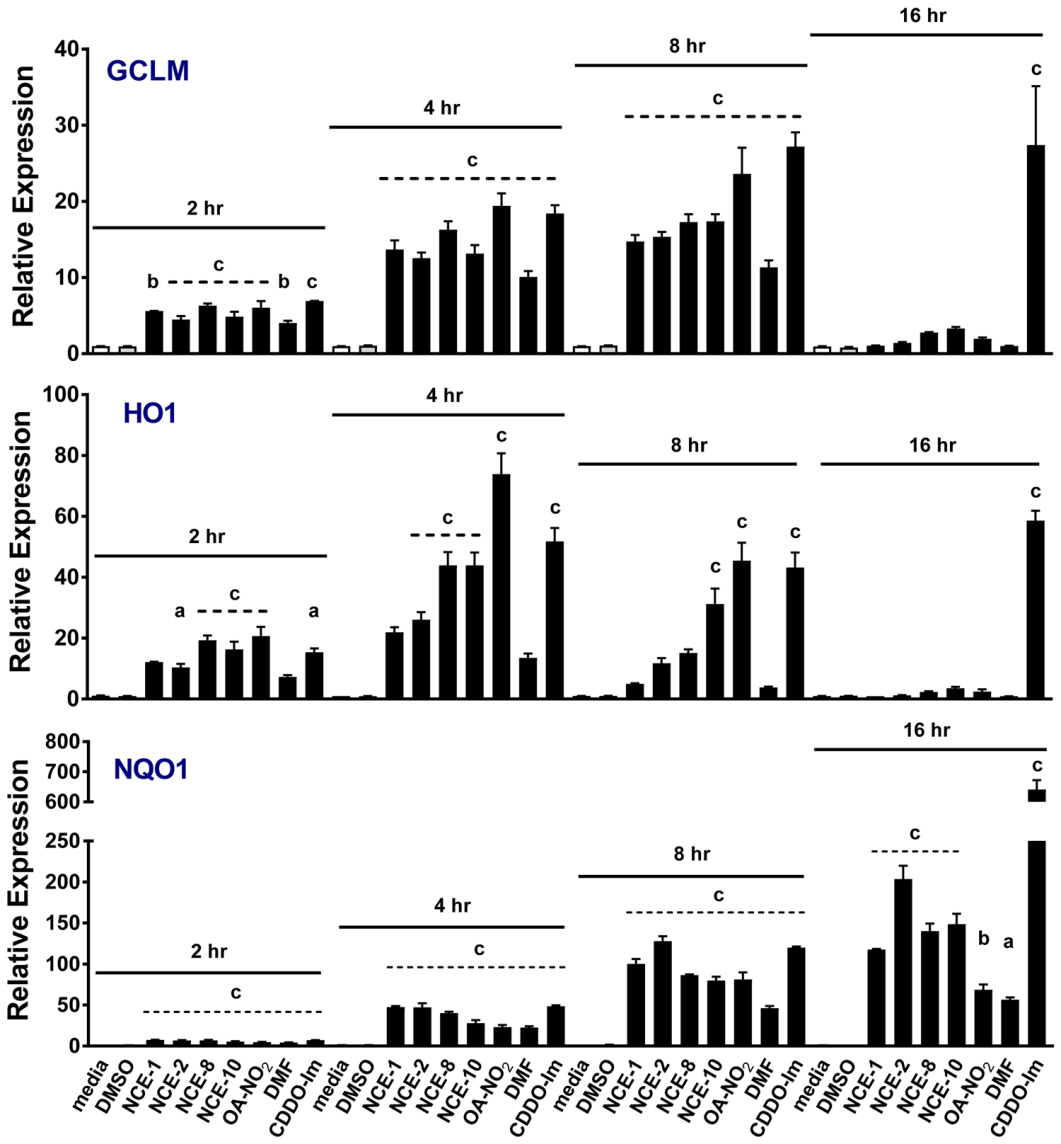
Time course of GCLM, HO1 and NQO1 steady-state RNA levels following treatment of RAW264.7 cells with electrophilic compounds. qPCR analysis showing relative mRNA levels following the treatment with 10 μM NCE-1, -2, -8, -10, OA-NO_2_, DMF and 100 nM CDDO-Im for 2, 4, 8 and 16 hr in RAW cells. The values in the bar graph represent mean ± SEM for at least three independent experiments (n = 3–12). Compared to the vehicle (DMSO) treatment group at each time point, statistical significance is indicated by: (a) p < 0.05; (b) p < 0.01 and (c) p < 0.001.

To determine whether luciferase-based responses to NCEs correlated with pharmacologically-relevant downstream signaling events, qPCR and Western blot analyses were performed. Raw 264.7 cells were treated with 10 μM NCE 1, 2, 8, 10, OA-NO_2_, 10 μM DMF or 100 nM CDDO-Im for 2, 4, 8 and 16 hr (Fig. 5, time course). In all treatments with Raw264.7 cells, there was no observed cytotoxicity determined by MTT and Calcein AM assays (not shown), similar to the stably-transfected cells (Tables 3 and 4). The steady-state mRNA levels of GCLM were significantly elevated by 2 hr and peaked between 4 and 8 hr for all electrophilic compounds tested. By 16 hr, all Nrf2-dependent responses to electrophilic compounds returned to baseline and were not significantly increased compared to the control, except for 100 nM CDDO-Im (>20-fold increase). Likewise, the electrophilic compounds induced a similar response for HO1, with the exception that 10 μM NCE-1 and DMF increased HO1 mRNA levels but did not reach statistical significance at 4 and 8 hr. Again, by 16 hr HO1 steady-state mRNA levels were similar to that of the control or vehicle (DMSO) treated RAW264.7 cells, except 100 nM CDDO-Im (>58-fold). As for NQO1 mRNA levels, there was a steady and significant increase compared to media and vehicle control (p < 0.001) between 8–16 hr for most of the electrophilic compounds and the responses remained elevated at 24 hr (not shown). This response differed from HO1 and GCLM, where mRNA levels returned to baseline at 16 hr, whereas NQO1 levels still remained elevated. A similar extended response for CDDO-Im was observed for NQO1 at the 16 hr time point, as previously shown for HO1 and GCLM, although the magnitude increase in mRNA levels was much greater (640-fold vs media control, p < 0.001). Both OA-NO_2_ and DMF responses peaked before 16 hr but were still statistically significantly different from the media and vehicle controls.

**Figure 6:**
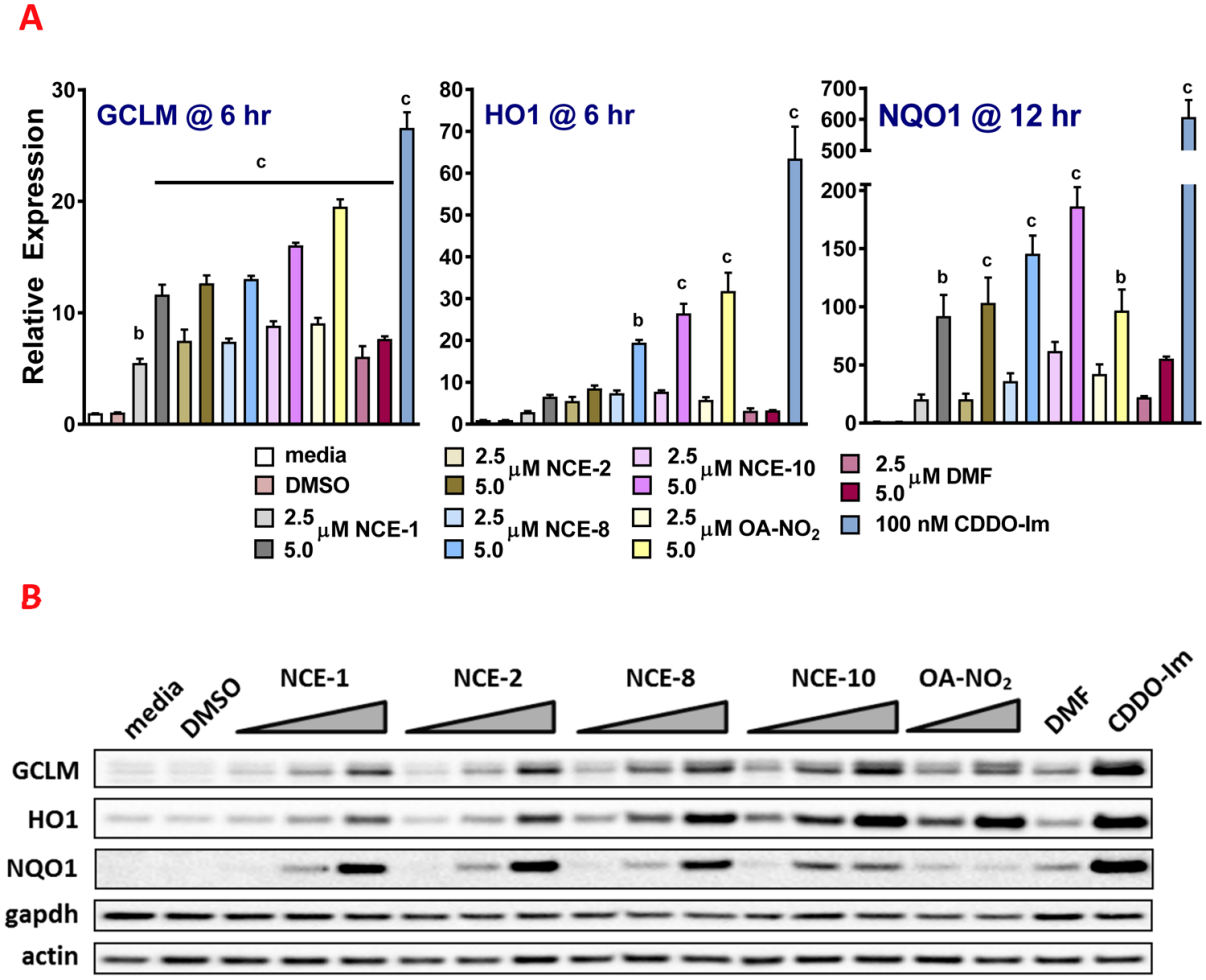
NO_2_-FAs increase Nrf2-dependent genes GCLM, HO1 and NQO1 in RAW264.7 cells. After the peak time point for the steady-state mRNA levels of GCLM, HO1 and NQO1 were determined, a dose response (2.5 and 5 μM) was performed. Dose-dependent increase in steady-state mRNA for GCLM and HO1 at 6 hr and NQO1 at 12 hr for nitroalkene derivatives, DMF and 100 nM CDDO-Im (**A**). Protein expression levels of GCLM, HO1 and NQO1 following treatment with electrophilic compounds (2.5–10 μM for NCEs, 5 and 10 μM OA-NO_2_, 10 μM DMF and 100 nM CDDO-Im) for 18 hr (representative Western blot, (**B**) Uncropped membranes with superimposed protein ladder (Bio-Rad, Precision Plus Protein Dual Color Standards, cat# 161–0374) are presented in Supplementary Figure 1. Within an individual experiment, there was a minimum of an n = 2/treatment which was normalized to the media alone treatment (cell media was replaced only). These technical replicates were averaged for each treatment. The values in the bar graph represent mean ± SEM for at least three independent experiments (n = 3−12). Compared to the vehicle (DMSO) treatment group at each time point, statistical significance is indicated by: (a) p < 0.05; (b) p < 0.01 and (c) p < 0.001.

After the peak time point for maximal steady-state mRNA expression of GCLM, HO1 and NQO1 were determined, a dose response (2.5 and 5 μM) was performed (Fig. 6A) using RAW264.7 cells. There was a dose-dependent increase in steady-state mRNA for GCLM and HO1 at 6 hr and NQO1 at 12 hr for NO_2_-FA derivatives, DMF and 100 nM CDDO-Im. For GCLM, there was a significant increase in response to all of the electrophilic compounds at concentrations of 2.5 and 5 μM for 6 hr ranging from 5.5-fold for 2.5 μM NCE-1 (p < 0.01 to DMSO) to 26.6-fold for 100 nM CDDO-Im (p < 0.001 to DMSO). There was a dose-dependent increase in HO1 mRNA induced by every electrophilic compound, with statistical significance being reached at the 10 μM concentration for NCE-8, NCE-10, OA-NO_2_ and 100 nM CDDO-Im (p < 0.001). The peak NQO1 steady-state mRNA expression was approximately 12 hr, all electrophiles at the 5 μM concentration reached statistical significance except DMF. The lower concentration of 2.5 μM for NCE-1, -2, -8, -10, OA-NO_2_ and DMF treatment increased fold activity above media control 20.3-, 20.3-, 36.0-, 61.9-, 42.1-, and 19.4-fold, respectively. The RAW264.7 cells responded after a 12 hr treatment of 100 nM CDDO-Im by increasing NQO1 steady-state mRNA levels greater than 600-fold (p < 0.001 vs media or DMSO treatment).

The protein expression levels of GCLM, HO1 and GCLM were also robustly increased in a dose-dependent manner following a 20 hr treatment with the NCEs -1, -2, -8 and -10. The fold increase of the NCE-induced protein expression, compared to media- or vehicle (DMSO)-treated RAW cells, was similar to that induced by OA-NO_2_, DMF and CDDO-Im (Fig. 6B).

**Figure 7:**
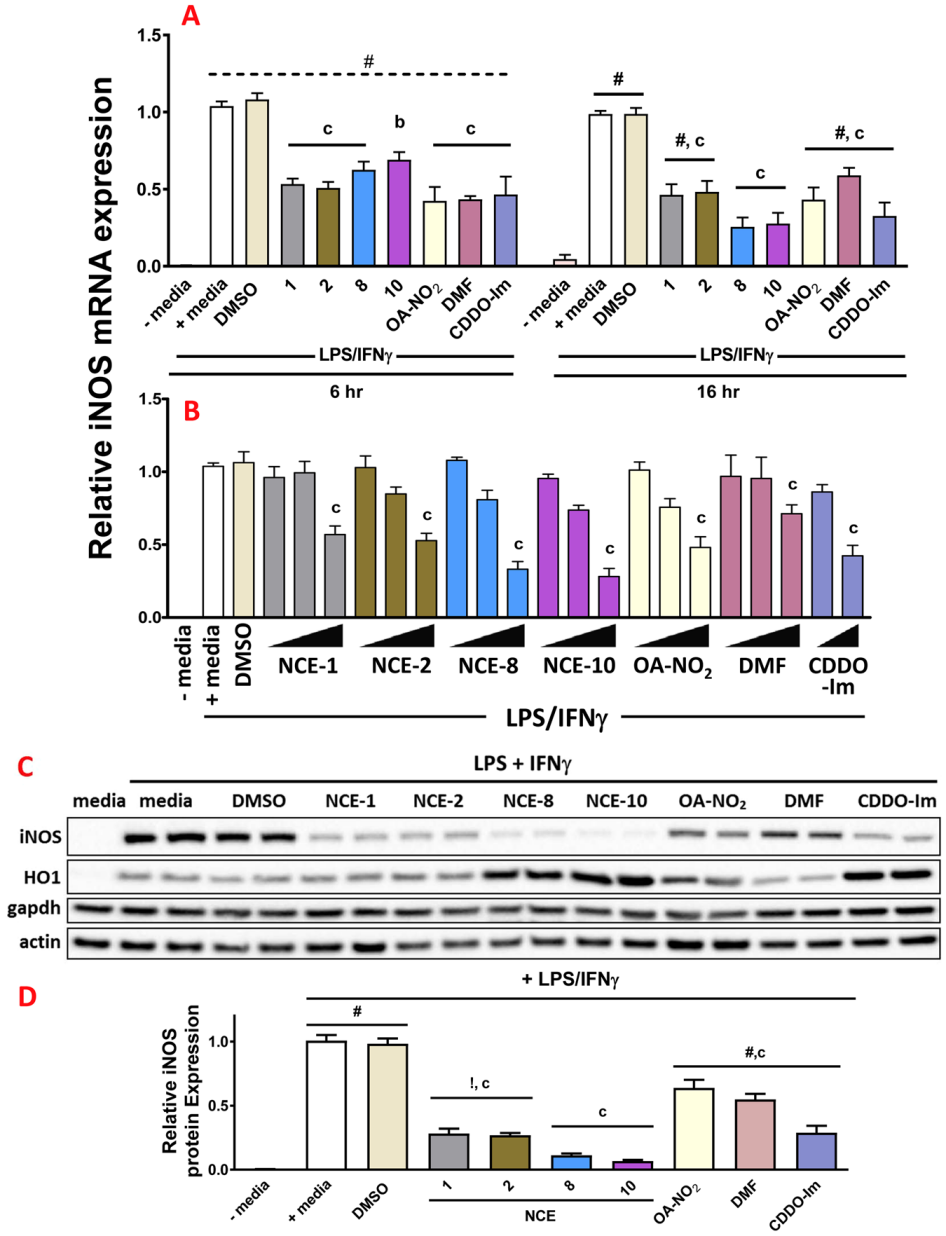
NO_2_-FAs inhibit LPS/IFNγ-mediated inflammatory markers iNOS in RAW264.7 cells. Raw cells were treated with 10 μM NCE-1, -2, -8, -10, OA-NO_2_, DMF or 100 nM CDDO-Im for 6 and 16 hr. Expression of iNOS mRNA was determined at 6 hr (**A**, left) and 16 hr (**A**, right). Raw cells were treated with 2.5, 5 and 10 μM NCE-1, -2, -8, -10, OA-NO_2_, DMF or 10 and 100 nM CDDO-Im for 16 hr and expression of iNOS mRNA was determined (**B**). Raw cells were treated with 5 μM NCE-1, -2, -8, -10, OA-NO_2_, DMF or 100 nM CDDO-Im for 20 hr. Representative Western blot of iNOS, HO1, actin and gapdh protein expression levels following treatment with electrophilic compounds for 20 hr (**C**). Uncropped membranes with superimposed protein ladder (Bio-Rad, cat# 161–0374) are presented in Supplementary Figure 2. Densitometric analysis was performed using housekeeping protein gapdh (**D**). Within an individual experiment, there was a minimum of an n = 2/treatment which was normalized to the media alone treatment (cell media was replaced only). These technical replicates were averaged for each treatment. The values in the bar graph represent mean ± SEM for at least three independent experiments (n = 3–12). ^#^p < 0.001 and ^!^p < 0.01 indicates statistically significant over media alone treatment without LPS/IFNγ at each time point; Compared to the vehicle (DMSO) treatment group at each time point, statistical significance is indicated by: (a) p < 0.05; (b) p < 0.01 and (c) p < 0.001.

The NCE 1, 2, 8 and 10 similarly inhibited LPS/IFNγ-induced inflammatory mRNA marker iNOS. The treatment of RAW264.7 cells with LPS/IFNγ induced a robust significant inflammatory response with a greater than 480-fold increase in iNOS expression at 6 and 16 hr (Fig. 7) (^#^p < 0.001). The co-treatment of all NCEs, OA-NO_2_, DMF (all at 10 μM) and 100 nM CDDO-Im with LPS/IFNγ induced significant inhibition of iNOS expression compared to LPS/IFNγ treated with either media alone or vehicle (DMSO) at 6 and 16 hr. Following the co-treatment of NCE-8 or NCE-10 with LPS/IFNγ at 16 hr, iNOS mRNA expression levels were 0.25 ± 0.06 and 0.28 ± 0.07 compared to LPS/IFNγ (media). Similar results were observed with a strong inhibition of MCP1 mRNA expression at 6 hr (not shown). RAW cells were then treated with increasing concentrations (2.5, 5 and 10 μM) of the electrophilic compounds noted above, except for 10 and 100 nM CDDO-Im, and iNOS mRNA expression was determined (Fig. 7B). Then, the electrophilic compounds were co-treated for 20 hr with the LPS/IFNγ inflammatory stimulus. The iNOS protein expression responses mirrored the 16 hr mRNA levels, with NCE-8 and NCE-10 the most effective at inhibiting this inflammatory response (Fig. 7C). The LPS/IFNγ inflammatory stimulus increased HO1 protein expression over untreated RAW cells. Moreover, both NCE-8 and NCE-10 robustly induced HO1 protein expression compared to vehicle-treated cells following the LPS/IFNγ stimulation.

## Discussion

After decades of rejecting new drug candidates that were either covalent modifiers or those that yielded electrophilic metabolites, both drug design and FDA approval strategies related to covalent drugs have recently done an about face^18,19^. For example, the FDA has recently approved the covalent modifiers afatanib, ibrutinib and osimertinib. We also now appreciate that aspirin, penicillin and, more contemporarily, omeprazole and clopidogrel act via covalent inhibition of their molecular targets^20^. At present, one of the most successful drugs for treating multiple sclerosis is DMF, an electrophilic oral drug noted for its ability to a) inhibit pro-inflammatory NF-κB signaling by alkylation of Cys38 in the p65 subunit^21^ and b) activate Nrf2-dependent gene expression by alkylating the Cys151 of the Keap1 regulator for Nrf2 signaling^22^. In the past, small molecule and biologics-based drug development has typically focused on strategies that direct a single molecule to a single target (e.g.-receptor, ion channel, enzyme) via drug-target interactions that occur under equilibrium conditions. In contrast, covalent inhibitors such as electrophilic fatty acids have the potential to display uniquely favorable pharmacokinetics and pharmacodynamics by targeting both catalytic nucleophiles of enzymes and functionally-significant nucleophiles of transcriptional regulatory proteins. This translates to advantages such as limited systemic exposure and conferral of greater potency, prolonged pharmacologic actions and a reduced risk for tolerance. Moreover, the complex metabolic, pulmonary, cardiovascular and renal diseases that are often comorbidities of modern problems such as obesity have proven to be resistant to therapeutic approaches based on single-target drugs. Consequently, patients are prescribed multiple single target drugs for treating complex problems that require the modulation of diverse signaling pathways.

The activation of Nrf2-dependent gene transcription of antioxidant and detoxification enzymes protects against oxidative stress, inflammation and drug toxicity. Activation of Keap1/Nrf2 signaling exerts beneficial effects in numerous disease states including atherosclerosis, diabetes, liver injury, malaria, obesity, neurodegenerative diseases and certain cancers^23–25^. Keap1 functions as a negative regulator of Nrf2 by binding to it in the cytoplasmic compartment, promoting ubiquitination and downstream degradation by the proteasome. Under metabolic and inflammatory stress conditions, typified by elevated levels of oxidizing and electrophilic species, specific Keap1 Cys residues are modified stabilizing the Keap1-Nrf2 complex, resulting in the accumulation of Nrf2 in the nucleus and transactivation of Nrf2 signaling. There are unique patterns of Keap1 thiol modifications induced by different classes of reactive species that in turn exert distinct biological responses^26^. Several critical Cys residues can be modified, the three most readily alkylated residues (Cys151, Cys273 and Cys288) are essential for Keap1 function^27^. Cys273 and Cys288 account for approximately 50% of OA-NO_2_ reactions; thus OA-NO_2_ is termed “Cys151 independent”, as the mutation of Cys151 of Keap1 has no effect on ARE activation^28^. Hence, OA-NO_2_ is classed as a Cys273/Cys288 inducer^24^, even though other Cys residues contribute to Keap1 function^28^. DMF preferentially alkylates Cys151. There are conflicting reports and limited insight as to the Keap1 Cys reactions that account for for the potent Nrf2 pathway activation that is induced by CDDO^24,29–31^.

For the present Keap1/Nrf2 luciferase activation studies, the three top NO_2_-FA inducers were NCE-10, -8 and -5, that all shared the same chemical profile as omega-12 nitro-alkenes (12 carbons from omega end to the double bond). These studies also revealed that both the α and ω acyl chain lengths modulate the potency of Nrf2 activation, as nitroalkene-containing alcohols and omega terminus nitroalkenes had no impact on modulating Keap1/Nrf2 (or NF-κB luciferase) activity.

Nitro-fatty acids inhibit multiple aspects of NF-κB signaling. Previous studies have shown that the mechanisms accounting for NF-κB signaling inhibition by OA-NO_2_ are multifaceted, as OA-NO_2_ (a) inhibits IKKβ preventing the degradation of the inhibitor IκBα, (b) alkylate the NF-κB RelA protein at Cys38 to prevent DNA binding and (c) promote RelA polyubiquitination and proteasomal degradation^32^. The proteolytic degradation of NF-κB subunits is also important in the termination of NF-κB activation. For example, RelA protein is regulated by ubiquitin- and proteasome-dependent degradation signals that terminate NF-κB activation^33–36^. Thiol-alkylating and S-nitrosating agents also can induce NF-κB subunit p50 degradation *via* PTM of Cys62^37^. The top two inhibitors of NF-κB luciferase activity following TNFα stimulation were NCE-1 and -2. Both compounds were omega-5 nitro-alkenes (5 carbons from omega end to double bond), highlighting a structural feature that maximizes the inhibition of NF-κB over other related fatty acid nitroalkenes. In most cases, as the number of carbons between the omega end and the nitroalkene increased, the ability to inhibit TNFα-induced NF-κB significantly decreased.

Are the luciferase screens good predictors of downstream signaling? The ARE luciferase screen was a strong indicator of Nrf2-dependent downstream signaling. NCE-10 had the highest ARE luciferase activity coupled with the most potent increases in GCLM, HO1 and NQO1 mRNA (at the optimal time point) and protein expression. However, the NF-κB screen revealed that NCE-1 and -2 were the most potent inhibitors. Yet, NCE-8 and -10 inhibited LPS/IFNγ-mediated iNOS mRNA and protein expression to a greater extent than NCE-1 or -2. For the most part, the NF-κB screen was a good predictor of the ability of the NCEs to inhibit LPS/IFNγ-mediated iNOS gene expression with one exception (NCE-8). The apparent discrepancy observed between the reporter assay and the inhibition observed in murine macrophages might be related to the regulation by Nrf2 of the NF-κB pathway previously demonstrated using genetic and pharmacological models^38^.

There is cross-regulation of Nrf2 and NF-κB signaling. In cell culture models, there is enhanced IKKβ activity and increased IκBα phosphorylation in Nrf2^−/−^ MEFs^39^. Also, sulforaphane, an isothiocyanate found in cruciferous vegetables, inhibits LPS-induced pro-inflammatory TNF-α and iNOS gene expression in primary peritoneal macrophages and this effect is lost in primary peritoneal macrophages devoid of Nrf2^40^. Moreover, excess Keap1 directly interacts with IKKβ and blocks TNFα-stimulated NK-κB activation^41^ suggesting that in cells without Nrf2, the cytoplasmic repressor Keap1 is playing a major role in NF-κB inhibition. There are additional proteins, besides Keap1 that regulate both Nrf2 and NF-κB directly. Some of these adaptor proteins include p62, MafK and β-transducin repeat containing E3 ubiquitin protein ligase, although the exact mechanisms are unclear. In this case, these electrophilic NCEs hit multiple pathways and the ability to specifically determine the exact molecular target and the pecking order will be difficult especially considering the evidence described above in Nrf2^−/−^ cells. With electrophilic signaling mediators, one must be aware that multiple pathways are targeted by these pleiotropic species. One must realize that all coins have two sides and that the broad reactivity pattern of electrophilic molecules may induce innocuous or even adverse signaling pathways. In this regard, preclinical studies using electrophilic NO_2_-FAs show no adverse effects at the dosing levels used to achieve beneficial pharmacological effects.

There are discrete chemical differences between NO_2_-FAs, electrophilic fatty acid metabolites and drug candidates in terms of reactivity, reversibility and molecular targets. For example, the second order rate constant for NO_2_-FA reaction with GSH (~170–355 M^−1^ sec^−1^) is 250–500 faster than other electrophilic fatty acid derivatives such as cyclopentenones (15-deoxy-prostaglandin-J_2,_ 15d-PGJ_2_, ~1 M^−1^ sec^−1^ and 8-iso prostaglandin *A2*, ~1 M^−1^ sec^−1^), α,β-unsaturated ketone [4-hydroxynonenal (HNE), ~1.3 M^−1^ sec^−1^) and for context, also hydroperoxides (the redox signaling mediator H_2_O_2_, ~1 M^−1^ sec^−1^)^4,42,43^. For electrophilic fatty acid-mediated PTMs to manifest non-pathogenic signaling responses and represent a potentially safe drug strategy, the reversibility of protein-electrophile adduction is important^44^. This property will (a) limit protein adduct accumulation, (b) avoid hyper-activation of signaling networks and (c) prevent the depletion of cellular GSH pool^44^. For these reasons, electrophilic cyclopentenone prostaglandins and aldehydic lipid oxidation products (e.g., 15d-PGJ_2_ and 4-HNE), which do not readily dissociate from sites of adduction, induce toxicity at low concentrations^45–50^. The unique resonance structure of fatty acid nitroalkene substituents confers both rapid Cys alkylation and NO_2_-FA dissociation (K_eq_ = 7.5 × 10^−6^) that restores unmodified Cys and electrophilic nitroalkene upon dissociation^51^. After ultimate reaction with highly abundant low molecular weight thiols such as Cys or GSH, NO_2_-FA adducts will be exported from cells via multi-drug resistance transporters and eventually excreted^51–53^. Preclinical and human studies affirm that these properties confer OA-NO_2_ with favorable pharmacokinetics and no apparent toxicity at concentrations well above predicted therapeutic levels (NCT: 02127190, 02248051, 02460146, 02547402 and 02313064). For Bardoxolone, DMF and MMF, the second order reaction rate constants and potential reversibility of reaction with thiols such as Cys or GSH have not been reported.

Spatial orientation and distance from electrophilic moiety drives function. Perhaps the best studied electrophile using structure-function approaches is CDDO. Previous studies determined that three rings (A, B and C) were responsible for the robust activity of the pentacyclic triterpenoid CDDO. More specifically, the cyanoenone in ring A and enone in ring C of CDDO form a double Michael acceptor to induce the Keap1/Nrf2 pathway and its potent anti-inflammatory activity^54–56^. A subsequent study using monocyclic cyanoenones, revealed that ring C has greater electrophilic potential than ring A. However, the addition of ring B allows the two electrophilic centers on rings A and C to have a specific spatial orientation and distance from one another to achieve maximal potency of CDDO (as well as the acetylenic moiety at the junction at rings B and C)^57^. Overall, these studies have confirmed that the chemical scaffold, the electrophilic group and the number of electrophilic groups are important determinants of Nrf2 activation. In this regard, our study showed a strong correlation between position of the nitroalkene group and activity. In particular, the three most potent activators of Nrf2 were omega-12 NCEs. Among this group, NCE-10 (contains 8 carbons from the nitroalkene to the carboxylic acid) had the highest ARE luciferase activity and was the most potent Nrf2 activator, as indicated by GCLM, HO1 and NQO1 gene expression. NCE-10 was followed by NCE-8 and NCE-5 each having 7 and 6 carbon long acyl chain on the α end respectively. In general, our study shows that as the omega end acyl chain length increases, the ability to induce activation is greater, pointing to a possible stabilization by hydrophobic interactions of the NCEs with Keap1. However, the results observed for NF-κB inhibition revealed that NCEs with shorter omega end acyl chains were more effective inhibitors. NCE-17 is a nitrated alcohol and is an inert electrophile in being able to alter ARE or NF-κB luciferase activity. These findings suggest that specific spatial orientation and distance of the electrophilic moiety plays a role in determining Nrf2 activation and NF-κB inhibition. This information is particularly interesting in guiding the selection of an electrophilic fatty acid for pre-clinical testing for indications where only Nrf2 or NF-κB modulation might be desired. However, it is also important to appreciate that these pleiotropic NCE can modulate other pathways beyond just Nrf2 or NF-κB.

Protein targets of electrophilic fatty acid nitroalkenes include an array of thiol-dependent stress sensor and stress response mechanisms. Proteomic analysis and targeted genetic approaches (mutagenesis, knockdown and over-expression) have identified functionally-significant and highly reactive Cys residues, sensitive to both oxidation and alkylation, in proteins that mediate inflammation and stress-responses. This includes the NO_2_-FA–responsive transcription factors NF-κB/p65, Keap1/Nrf2, PPARγ and heat shock factor-1, as well as enzymes and ion channels such as xanthine oxidoreductase, NADPH oxidases, soluble epoxide hydrolase, 5-lipoxygenase and the transient receptor potential ion channel family^10,28,58–68^. Electrophilic lipid-mediated adaptive responses to stress also cross species boundaries, being important in plants^69^. More importantly, some of these pathways observed in plants may be conserved in humans. With these highly conserved gene regulatory and systemic response mechanisms NO_2_-FAs act as pleiotropic signaling mediators that both induce and suppress the expression of >500 genes in human cells^8^. In aggregate, these reactions will regulate systemic adaptation to pathogenic stimuli and loss of metabolic homeostasis.

Finally, NO_2_-FAs provide an important lipid-based pharmacologic approach that capitalizes on a nucleophile-targeted drug strategy that can favorably impact multiple signaling pathways and metabolic events. Oxidative stress and inflammation are underlying hallmarks for almost any disease state. Being able to devise a drug, such as electrophilic NO_2_-FAs, that will induce a robust antioxidant response and potently inhibit inflammatory mediators without undue side effects, will be useful for treating diseases that are unresponsive to single target drugs. Risk is managed in part by the use of an endogenous mediator class (fatty acid nitroalkenes) and the knowledge that there are already >40 highly effective FDA-approved covalent drugs that have both a storied past and a promising future^20^. Single molecules, such as lipid electrophiles, that display pleiotropic poly-agonist and poly-inhibitor qualities, have the potential to be much more effective in treating clinical problems having a multi-factorial pathogenesis. In other words, a drug strategy that is directed towards inducing PTMs of the redox-responsive thiol proteome is anticipated to strike a chord, rather than a note, in treating complex disease phenotypes.

## Methods

### Materials

All materials were reagent grade and purchased from Sigma unless otherwise specified.

### Cell culture

Murine RAW264.7 macrophages were obtained from American Type Culture Collection (Manassas, VA), maintained in complete media (DMEM containing 10% FBS, 100 U/ml penicillin and 100 μg/ml streptomycin) at 37 °C in 5% CO_2_. RAW264.7 cells were seeded the night before treatment. The next day, solutions in complete media were prepared and treated for the corresponding time points. The ‘media’ group only had the media replaced at the time of treatment (media sham). The vehicle used to solvate all of the fatty acids was DMSO and this group contained <0.1% DMSO (v/v) final concentration.

### New chemical entities

The NCEs were synthesized by Piramal Healthcare, India using commercially available fatty acids and fatty alcohols as starting materials and a nitroselenation reaction^70^. Stock concentrations of NCEs were solvated in dimethylsulfoxide (DMSO) and kept at −80 °C. The products were purified and the structures of all NCEs confirmed by ^1^H NMR, chromatographically by HPLC-mass spectrometry (LC-MS)(Table 2).

### Chromatography

LC-MS analysis of NCEs employed a C18 reverse phase Luna column (length 100 mm, 5 µm particle size, 2.0 mm inner diameter from Phenomenex) at a 0.65 ml/min flow rate of a binary solvent system consisting of water +0.1% acetic acid (solvent A) and acetonitrile +0.1% acetic acid (solvent B) to resolve the individual NCEs. The gradient was set as follows: 20% B (1 min) and NCE eluted with a linear increase in solvent B to 100% over 10 min.

### Mass spectrometry

NCEs eluting from the HPLC were directly analyzed on an API4000 Qtrap triple quadrupole mass spectrometer (Applied Biosystems, San Jose, CA) equipped with an electrospray ionization source. The first quadrupole was set to filter the mass corresponding to the negatively charged NCE. NCEs were monitored via the specific formation of an NO_2_^−^ ion upon collision-induced fragmentation. In addition, purity and composition for all compounds were confirmed by HPLC-MS on a high resolution mass spectrometer on an LTQ Velos Orbitrap (Thermo Scientific) equipped with a HESI II electrospray source. The following parameters were used: source temperature 450 °C, capillary temperature 360 °C, sheath gas flow 20, auxiliary gas flow 15, sweep gas flow 3, source voltage 3.75 kV, S-lens RF level 68 (%).

### Spectrophotometry

NCE were dissolved in DMSO at a final concentration of 15 μM and an absorbance spectrum was obtained between 200 and 400 nm. NCE stocks (20 mM) were periodically evaluated (every two weeks) to confirm stability of stock solutions. Concentrations were calculated in buffer phosphate using an ε_268_ = 8.22 M^−1^ cm^−1^ as previously reported^4^.

### Luciferase reporter analyses

The ARE reporter cell line contains a firefly luciferase gene under the control of an ARE stably integrated into HepG2 cells. The NF-κB reporter cell line also contains a firefly luciferase gene (driven by 4 tandem repeats of the NF-κB response element located upstream of the minimal TATA promoter) and stably integrated into HEK293 cells. Both luciferase reporter cells were purchased from BPS Bioscience (San Diego, CA) and were cultured according to BPS Bioscience’s instructions with one minor exception (cells were grown in 20% FBS medium immediately after they were thawed out of liquid nitrogen). The media was then changed to 10% FBS after the first passage. Following treatment, cells were rinsed with phosphate buffered saline (PBS), lysed with Passive Lysis Buffer (Promega) and luciferase activity was measured using Luciferase Assay System (Promega) according to manufacturer’s instructions. The ARE experimental design involved seeding stable ARE-luc HepG2 cells in the afternoon (day 0). Approximately 24 hr later, NCE treatments were prepared in complete medium (10% serum with the selective agent geneticin). Shortly after the NCE treatments were prepared, media was removed from wells and then the cells were treated overnight for 18 hr. On day 2, luciferase reporter assays were performed (see above). The relative ARE activity for all NCEs were normalized to the media alone treatment (only the media on the cells was replaced). The NF-κB experimental design involved stable NF-κB-luc Hek293 cells being seeded in the afternoon (day 0). Approximately 18 hr later, treatments with NCEs combined with a final concentration of 500 pg/mL TNFα were prepared in full medium (containing 10% serum with the selective agent Hygromycin B). Shortly after treatments prepared, media was removed from wells and then cells were treated for 2 hr. Following the 2 hr treatment, luciferase reporter assays were performed. Statistical significance was defined as p < 0.05 using the Tukey posthoc test following one-way ANOVA for all 27 treatments- 22 NCEs, OA-NO_2_, DMF, CDDO-Im, media and DMSO).

### Cell viability

Cytotoxicity/cell viability method were determined by the fluorescent Calcein AM and the colorimetric MTT [3-(4,5-dimethylthiazol-2-yl)-2,5-diphenyltetrazolium bromide] assays. Fluorescence of calcein was measured as previously reported^71^ and the MTT assay was determined as previously^72^. In brief, the viable cells convert the water soluble tetrazolium salt (yellowish solution) into a purple colored formazan product with an absorbance maximum near 570 nm. In all cell treatments, the final DMSO concentration was <0.1% (v/v) in complete media. Cell viability was normalized to the media alone treatment (without 500 pg/mL TNFα for inflammation studies).

### Western blotting

RAW264.7 cells were rinsed with ice-cold phosphate buffer saline (PBS) and homogenized in RIPA buffer. The cells were disrupted by sonication on ice and then centrifuged at 12,000 g for 5 min at 4 °C. The protein concentration was determined by the BCA protein assay kit (Pierce, Rockford, IL). Protein was denatured by boiling, resolved by SDS-PAGE, and transferred to nitrocellulose (BioRad, Hercules, CA). Membranes were probed with antibodies to inducible nitric oxide synthase (iNOS) at 1:1500 (Cell Signaling; 13120), heme oxygenase1 (HO1) at 1:5000 (Enzo Life; ADI-SPA-896), NAD(P)H dehydrogenase quinone 1 (NQO1) at 1:1500 (Abcam, ab34173), glutamate–cysteine ligase complex modifier subunit (GCLM) at 1:1000 (Invitrogen, 50–55–466) overnight, washed with TTBS, and then incubated with horseradish peroxidase-conjugated antibodies at 1:10,000 dilution. Immunoreactive bands were detected using chemiluminescence (BioRad). The antibody order for the Nrf2-dependent proteins was always GCLM, NQO1 and then lastly HO1. The HO1 antibody was very strong and difficult to completely strip immunoreactive bands from membrane. Control experiments were performed to ensure that there were no immunoreactive bands following the stripping protocol for GCLM and NQO1. To verify protein loading, membranes were subsequently stripped and reprobed with mouse monoclonal antibodies against β-actin (Sigma) as well as the human polyclonal glyceraldehyde 3-phosphate dehydrogenase (gapdh) purchased from Sigma (A4700) and Trevigen (2275), respectively.

### Quantitative real-time PCR

Total RNA was extracted from RAW264.7 cells using TRIzol reagent (Invitrogen, Carlsbad, CA, USA). RNA was reverse-transcribed using the iScript cDNA synthesis kit (BioRad, Hercules, CA, USA) as previously described^73^. Gene expression was determined by quantitative real-time (RT)-PCR (qPCR) using TaqMan gene expression assays-on-demand (Applied Biosystems, Foster City, CA, USA) and normalized to gapdh or actin using the comparative *C*_t_ method.

### Statistical analysis

Results are expressed as mean ± SEM. Statistical analysis was performed with the GraphPad Prism, and the data were analyzed by one-way analysis of variance (ANOVA) with Tukey’s multiple comparison *post hoc* comparisons and Student’s *t*-test. All results are considered significant at *p* < 0.05.

## Electronic supplementary material

Supplementary material
