# Supplementary material for: Electrophilic fatty acid nitroalkenes regulate Nrf2 and NF-κB signaling:A medicinal chemistry investigation of structure-function relationships
Source: Sci Rep. 2018 Feb 2;8:2295. doi: 10.1038/s41598-018-20460-8 (PMC5797128; doi:10.1038/s41598-018-20460-8)

Electrophilic fatty acid nitroalkenes regulate Nrf2 and NF-κB signaling: A medicinal chemistry investigation of structure-function relationships

Nicholas K.H. Khoo[1,*], Lihua Li[1], Sonia R. Salvatore[1], Francisco J. Schopfer[1,*] and Bruce A. Freeman[1,*]

[1] Department of Pharmacology and Chemical Biology, University of Pittsburgh, Pittsburgh, PA 15261

* To whom correspondence should be addressed. Email: nkhoo@pitt.edu (N.K.H.K), fjs2@pitt.edu (F.J.S.) and freerad@pitt.edu (B.A.F.)

Supplementary Material

Supplementary Figure 1. Immunoblots of GCLM, HO1, NQO1, gapdh and actin. Representative uncropped membranes with superimposed protein ladder (Bio-Rad, Precision Plus Protein Dual Color Standards, cat# 161-0374). Membranes were cut directly below the 37 kDa marker to probe for GCLM, HO1 and NQO1. The membrane above 37 kDa was probed for gapdh and actin.

Supplementary Figure 2. Immunoblots of iNOS, HO1, gapdh and actin. Representative uncropped membranes with superimposed protein ladder (Bio-Rad, Precision Plus Protein Dual Color Standards, cat# 161-0374). Membranes were cut horizontally directly above the 100 kDa marker to probe the top membrane for iNOS. The lower part of the membrane was probed for HO1, gapdh and actin. The gapdh membrane was stripped and reprobed after obtaining the HO1 signal. HO1 is a very strong signal and was not completely removed after stripping the membrane as indicated by `HO1.

Supplementary Figure 1

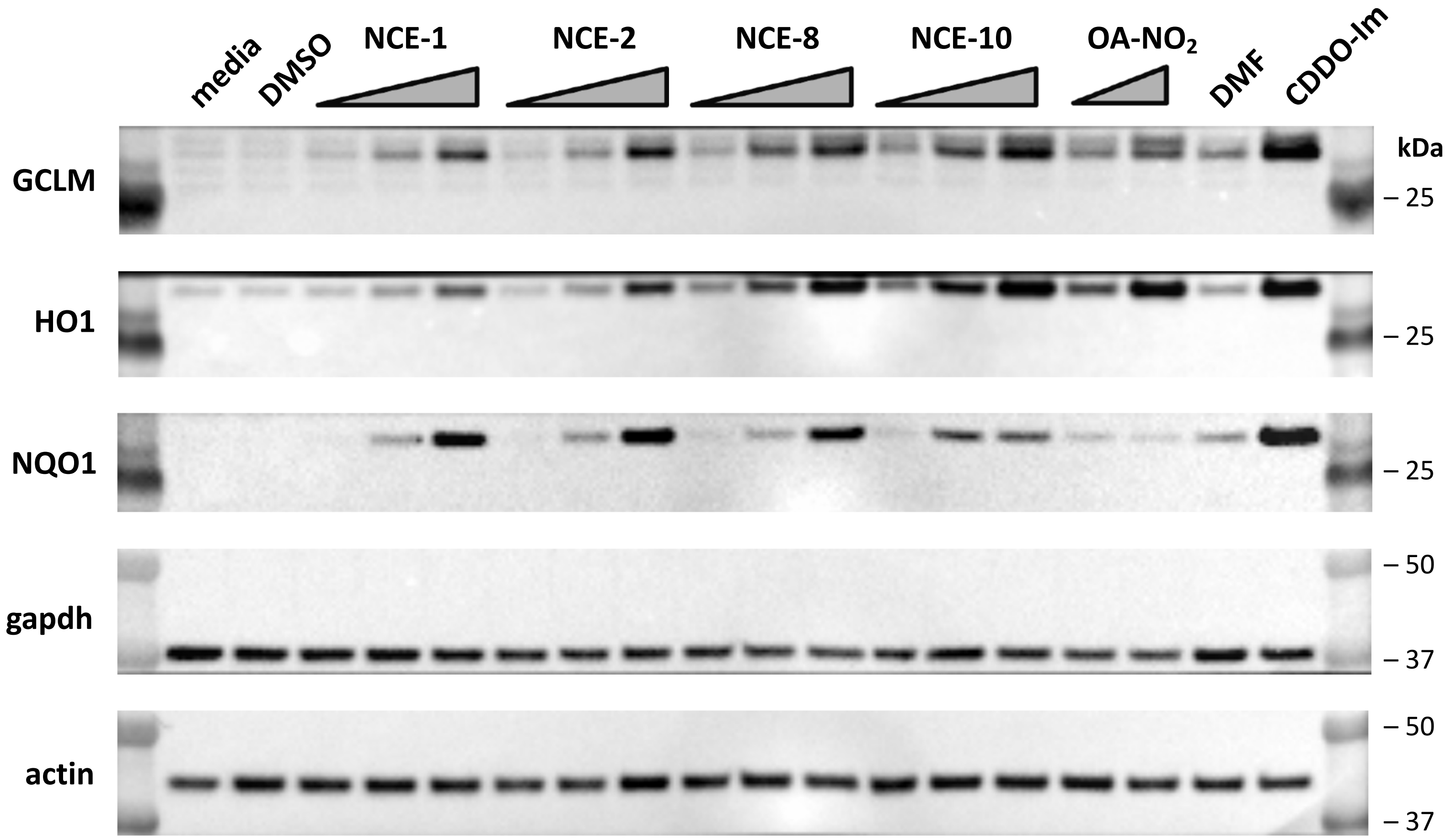

Supplementary Figure 2

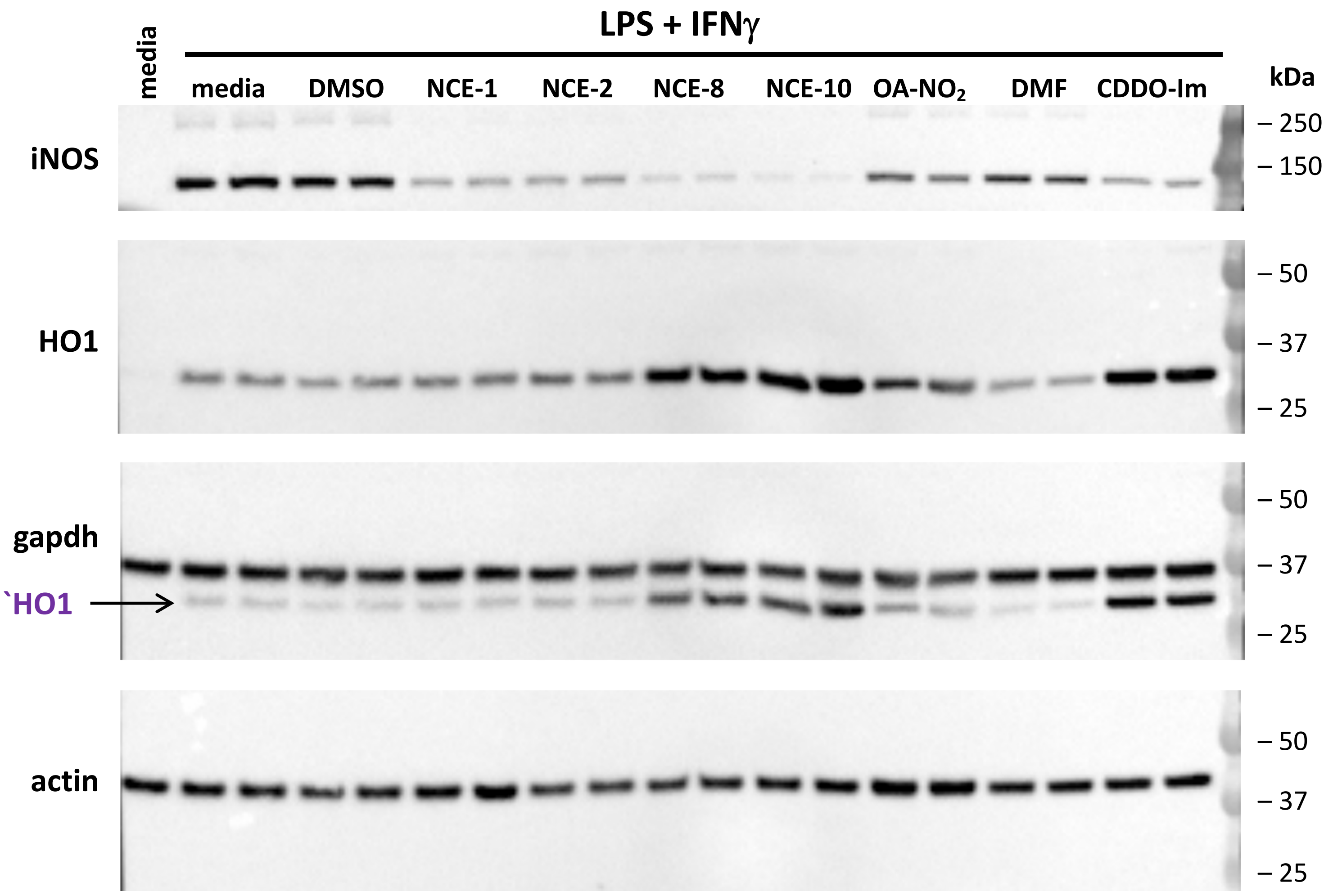

Supplement: Supplementary file 1 — Supplementary material [file 41598_2018_20460_MOESM1_ESM.pdf]
